# Effect of Cold Deformation and Heat Treatment on the Microstructures and Mechanical Properties of Au-15Ag-12Cu-6Ni Alloy Sheets

**DOI:** 10.3390/ma17020356

**Published:** 2024-01-10

**Authors:** Haodong Chen, Xinyue Cui, Songxiao Hui, Changheng Li, Wenjun Ye, Yang Yu

**Affiliations:** 1 State Key Laboratory of Nonferrous Metals and Processes, China GRINM Group Co., Ltd., Beijing 100088, China; chen5227239463@126.com (H.C.);; 2 GRIMAT Engineering Institute Co., Ltd., Beijing 101407, China; 3 General Research Institute for Nonferrous Metals, Beijing 100088, China; 4 College of Arts & Information Engineering, Dalian Polytechnic University, Dalian 116400, China; 5 GRINM (Guangdong) Institute for Advanced Materials and Technology, Foshan 528051, China

**Keywords:** gold alloy, dynamic recrystallization, texture evolution, wear resistance

## Abstract

The evolution of the microstructure and hardness changes in the Au-15Ag-12Cu-6Ni alloy during the processes of cold rolling and annealing were investigated and the heat treatment regimen for the alloy was optimized in this article. The hardness of the alloy continuously increases with the cold rolling reductions, leading to continuous deformation of the grains during the cold rolling process, ultimately resulting in smaller grain sizes. Subsequent annealing induces recovery and recrystallization, achieving complete recrystallization at 700 °C. An intriguing softening effect is observed after annealing at 700 °C, manifesting in a significant reduction in hardness to 238 (Hv_0.5_). The cold deformation texture of the alloy aligns with the recrystallization texture type, exhibiting only a certain degree of angular deviation. This is primarily characterized by <111>//RD texture and a texture deviating 60° from RD towards TD. The performance of the finished sheet improves with the precipitation of ordered phases AuCu after a 300 °C heat treatment for 0.5 h, resulting in a remarkable hardness of 380 (Hv_0.5_).

## 1. Introduction

With the rapid development of the aerospace, shipping and other industries, the requirements for the electrical contacting property, chemical performance, mechanical performance, and other aspects of conductive rings are continuously increasing. The demand for long-life conductive rings with a high strength, wear resistance, and corrosion resistance has increased significantly [1,2,3]. Among the array of precious metal electrical contact materials, gold stands out for its unrivaled chemical stability, commendable electrical conductivity, low elastic modulus, and remarkable wear resistance. However, pure gold is prone to adhesive welding, has low strength and hardness, a high coefficient of friction and significant susceptibility to wear. Addressing these challenges involves the addition of trace elements to fortify gold alloys through mechanisms such as solid solution strengthening, precipitation strengthening, grain refinement, and enhanced crystallinity [4,5,6]. Multi-element gold-based alloys such as AuAgCu, AuCuPt, and AuCuNi have been developed by combining traditional alloying elements with microalloying elements. These alloys exhibit superior elasticity, robust wear resistance, corrosion resistance, and low contact resistance. They find applications as elastic brush materials in precision potentiometers and as contact materials in miniature relays [3,5].

Gold alloys typically incorporate strengthening elements (Cu, Ag, Pd, Pt, Ni, Co) and resistance-sensitive elements (Fe, Mn, Cr, V) [7,8,9]. It is possible to enhance strength and adjust electrical resistance as needed by adding alloying elements. Gold-based conductive ring materials are primarily focused on the Au-Ag-Cu and Au-Ag-Cu-Ni alloy systems. In the Au-Cu alloy system, the formation of ordered phases such as AuCu and Au_3_Cu [4,5,10] can double the alloy’s strength and enhance the alloy’s wear resistance while substantially reducing its electrical resistance. [11]. For Au-Ni alloys, precipitation hardening contributes to a notable enhancement in properties, particularly wear resistance [12]. Fu et al. [13] studied the mechanisms of Ni addition to improve the mechanical properties of the Au-20Ag-10Cu alloy. Their research found that the addition of Ni effectively enhanced the solid solution strengthening effect of the Au-20Ag-10Cu alloy causing greater lattice distortion. The addition of Ni promoted the precipitation of the AuCu phase, inducing lattice distortion in the ordered phase, which contributed to precipitation strengthening. The addition of Ag to these alloys allows for solid solution strengthening while maintaining the alloy’s resistivity and thermal conductivity [14,15,16]. T. Shiraishi et al. [17] studied the impact of adding a small amount of silver to an equiatomic AuCu alloy via the ordered process of AuCuI and the aging hardening behavior at 300 °C. The results indicate that adding a small amount of silver to equiatomic AuCu helps stabilize the mechanical properties of the aged alloy.

The recrystallization behavior of cold-rolled sheet metal during the annealing process is closely related to the alloy’s performance. Analyzing the microstructure of alloys under different annealing conditions can deepen the understanding of the recrystallization mechanism. Wang et al. [18] investigated the effects of annealing on microstructure and properties of GH3536 superalloy sheets in detail. Their research indicated that the GH3536 superalloy exhibited a significant presence of low-angle grain boundaries (LAGBs) formed by dislocation motion and accumulation. After annealing at 950 °C, deformed grains were replaced by new strain-free grains during the recrystallization. S. Sadeghpour et al. [19] studied the influence of cold rolling and subsequent annealing on the microstructure and mechanical properties of the Ti-4Al-7Mo-3V-3Cr alloy. They also indicated that recrystallized grains often have a lower dislocation density. Increasing the annealing temperature or extending the annealing time facilitates the transformation of heavily deformed β grains with a high proportion of LAGBs into uniformly fine equiaxed grains. Zhao et al. [20] characterized the microstructure, macro-texture, grain orientation, and misorientation evolution of a cold-rolled Mg-Zn-Gd alloy during annealing at 150–480 °C. The study suggested that sub-grains are potentially recrystallized grains, as the annealing temperature rises. On the other hand, partially formed high-angle grain boundaries (HAGBs) were formed from 2–6° LAGBs to HAGBs with misorientation < 30° by climbing and the cross-slip of dislocations. There is little research on the recrystallization mechanism of gold-based alloys. Combing microstructural analysis with the optimization of mechanical properties is essential for accurately optimizing the process.

Improving the wear resistance, corrosion resistance, and service life, while lowering contact resistance, can be achieved by increasing the hardness of gold-based alloys. This article investigates the effects of cold rolling reductions and annealing temperature on the microstructure and hardness of the Au-15Ag-12Cu-6Ni alloy used for conductive rings. Additionally, the heat treatment regimen of the finished sheet was optimized with hardness as the target.

## 2. Materials and Methods

**Figure 1:**
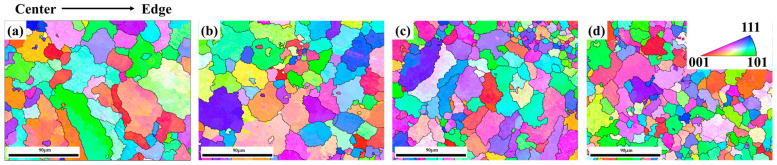
Microstructure of different positions of the Au-15Ag-12Cu-6Ni alloy ingot. (**a**) center; (**b**) near center; (**c**) near edge; (**d**) edge.

The ingot was produced by melting raw materials such as gold (99.99%), silver (99.99%), electrolytic nickel (99.99%), and electrolytic copper (99.99%) in a high-frequency vacuum induction furnace with the dimensions 33 mm × 10 mm × 61.6 mm. The chemical composition analysis results of the alloy ingot, conducted using energy dispersive X-ray spectroscopy (EDS), are presented in Table 1. The alloy compositions at different locations of the ingot show very minor differences indicating good uniformity in terms of composition. The coarse microstructure of different positions at the bottom of the original ingot is shown in Figure 1. The EBSD grain size statistics indicate that the grain size gradually decreases from the center to the edge of the ingot, with average grain sizes of 38 μm, 31 μm, 26 μm, and 21 μm, respectively. Therefore, homogenization annealing was carried out on the ingot to improve the unevenness in grain size.

The Au-15Ag-12Cu-6Ni alloy ingot was cold-rolled with different reductions of 20%, 40%, 60%, and 80% to investigate the cold deformation capacity. The cold-rolled samples will be called CR-40%, CR-60%, etc., hereafter, respectively. Subsequently, cold-rolled sheets subjected to the maximum reduction underwent annealing at temperatures of 550~750 °C for 0.5 h each to optimize the stress relief annealing process. The samples after stress relief annealing will be referred to as SRA-550 °C, SRA-600 °C, etc. After stress relief annealing, the sheet underwent the same deformation in cold rolling and was subjected to heat treatment optimization with heating at 300~600 °C, each for durations of 0.5~2 h, followed by air cooling. Additionally, after heat treatment at 750 °C for 0.5 h, a second heat treatment was conducted on the alloy with a temperature range of 200–500 °C for 0.5~2 h. The samples subjected to low-temperature annealing heat treatment and those subjected to double annealing heat treatment will be, respectively, referred as HT-300 °C/0.5 h and DHT-200 °C/0.5 h. In this article, carbon blocks were added to the furnace in all heat treatment processes to prevent the oxidation of ingots and sheets.

The Au-15Ag-12Cu-6Ni ingot and sheet were cut into 10 mm (along the rolling direction) × 8 mm samples for microstructural observation. Firstly, samples were ground up to 4000# by SiC sandpapers, then polished by diamond suspensions with particle sizes of 5 μm and 1.5 μm and finally polished by 0.04 µm Struers OP-S. The electron backscatter diffraction (EBSD) characterizations were carried out normal to the rolling direction by JEOL JSM-7900F with a step size of 6 µm at a voltage of 20 kV. EBSD data was analyzed and EBSD maps with low-confidence indexes were cleaned up by TSL-OIM software v7.3.1. A copper target for X-ray diffraction (XRD) was used to investigate the phase composition of the Au-15Ag-12Cu-6Ni alloy in the typical 2θ range of 10° to 90° with a scanning rate of 10°/min. The transmission electron microscope (TEM) observations were carried out by an FEI TECNAIG2 F20 microscope. The samples were initially ground with sandpaper to approximately 50 μm then were thinned down using the twin-jet polishing technique with a solution of 10 vol.% HClO_4_ and 90 vol.% C_2_H_5_OH.

The hardness of different conditioned samples was tested using an FM-ARS-9000. The samples were ground to 3000# with SiC sandpapers. During the hardness measurements, the load was 0.5 kg with a dwell time of 15 s and each sample was measured 5 times at different points.

## 3. Results and Discussion

### 3.1. The Impact of Cold Rolling Reductions on the Microstructure and Properties of the Au-15Ag-12Cu-6Ni Alloy

**Figure 2:**
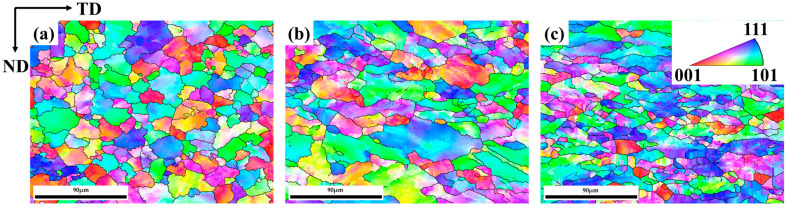
Microstructure of different cold rolling reductions: (**a**) CR-20%, (**b**) CR-40%, (**c**) CR-60%.

Gold alloys are known for their good cold deformation capability and the cold deformation capacity of the Au-15Ag-12Cu-6Ni alloy was assessed through rolling with different reductions of 20%, 40%, 60%, and 80%. Cracks appeared during the cold rolling at a thickness of 3.4 mm, indicating that the alloy had reached its cold-deformation limit, which was found to be 66%. The inverse pole figure (IPF) of the alloy under different rolling reductions is depicted in Figure 2. At a cold rolling reduction of 20% (Figure 2a), the alloy still exhibited a uniformly fine equiaxed microstructure with only minor deformation. As rolling reductions increased, grains continuously elongated along the rolling direction under the influence of force as seen in Figure 2b. Further increases in rolling reduction, reaching 60%, resulted in grains being further elongated and small grains appearing, as shown in Figure 2c. The increased cold rolling reduction caused larger grains to be fragmented along the subgrain boundaries.

**Figure 3:**
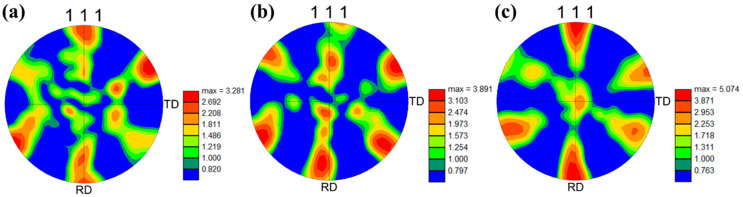
(111) Pole figures of the Au-15Ag-12Cu-6Ni alloy under different rolling reductions: (**a**) CR-20%, (**b**) CR-40%, (**c**) CR-60%.

Differences in cold rolling reductions often induce texture changes in various alloy systems [21,22,23]. In face-centered cubic (FCC) alloys, the <111> direction is typically one of the most stable crystallographic directions within the FCC structure, possessing high symmetry [24]. Figure 3 illustrates the (111) pole figures for the alloy at various cold rolling reductions. Overall, the texture types of the alloy do not change significantly with different cold rolling reductions. In the (111) pole figure, the cold-rolled alloy exhibits a texture primarily at the rolling direction (RD) pole point and around a deviation of approximately 60 degrees from the RD towards the transverse direction (TD). When the cold rolling reduction increases from 20% to 40%, the texture at the RD pole point slightly deviates towards the TD. The texture is similar to CR-20%, but a weak texture appears at the normal direction (ND) pole point when the cold rolling reduction increases to 60%. As the cold rolling reduction continues to increase, the texture strength increases from 3.281 to 5.074.

**Figure 4:**
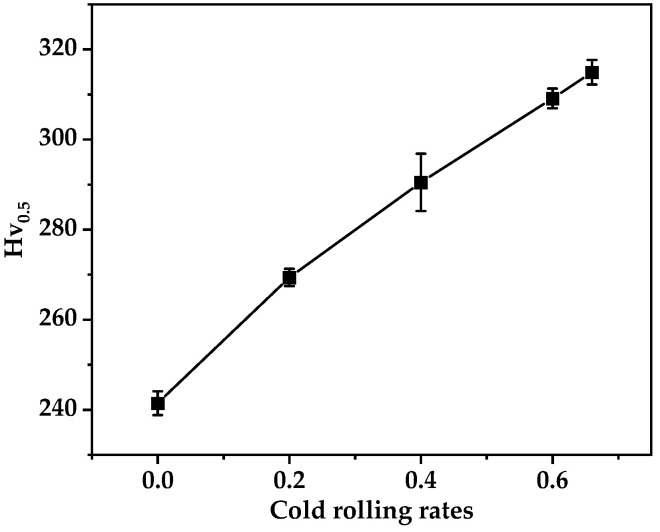
The trend in average hardness of the Au-15Ag-12Cu-6Ni alloy at different cold rolling reductions.

Different rolling reductions result in varying levels of texture strength leading to differences in the hardness of the alloy. The hardness of alloys with different rolling reductions was measured to study the effect of cold rolling reductions on alloy hardness. The change in average hardness of the Au-15Ag-12Cu-6Ni alloy with different cold rolling reductions is shown in Figure 4. The hardness gradually rises with the increase in cold rolling reduction. However, the reduction in hardness improvement gradually slows down as the cold rolling reduction increases. The final hardness increased from the as-cast state of 240 (Hv_0.5_) to 315 (Hv_0.5_) at 66% of the cold rolling reduction. The alloy displayed work-hardening characteristics with an accelerated increase in dislocation multiplication during the cold rolling process, contributing to the observed hardness increase [25,26]. The cold rolling significantly increases the hardness of the Au-15Ag-12Cu-6Ni alloy.

### 3.2. The Impact of Stress Relief Annealing Temperature on the Mcrostructure and Properties of Alloys

The Au-15Ag-12Cu-6Ni alloy demonstrates a significant cold work hardening effect. To facilitate further processing of the alloy, a stress relief annealing treatment is recommended. Annealing temperatures of 550 °C, 600 °C, 650 °C, 700 °C, and 750 °C, each with an annealing time of 30 min, were investigated on 66% cold-rolled samples. Subsequent to the annealing treatments, hardness testing and microstructure observation were conducted to study the influence of annealing temperature and determine the optimal stress relief annealing temperature.

**Figure 5:**
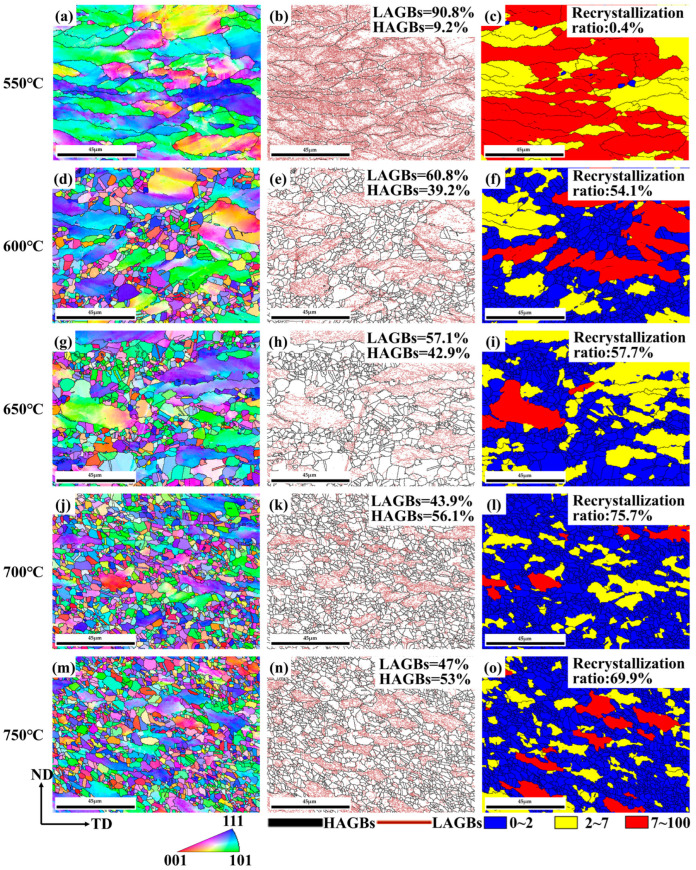
IPF maps, GB maps, and GOS maps at different annealing temperatures: (**a**–**c**) SRA-550 °C, (**d**–**f**) SRA-600 °C, (**g**–**i**) SRA-650 °C, (**j**–**l**) SRA-700 °C, (**m**–**o**) SRA-750 °C.

An analysis of the alloy microstructure at different annealing temperatures was conducted using EBSD. Figure 5 illustrates the IPF maps grain boundary orientation distribution maps (GB maps), and grain orientation spread maps (GOS maps) of the alloy’s microstructure at different annealing temperatures. The SRA-550 °C exhibits a typical deformation microstructure (Figure 5a). Almost all grains have been elongated along the rolling direction resulting in a coarse grain size. In Figure 5b, the red lines represent LAGBs with misorientation between 2 and 15° and the black lines represent HAGBs with misorientation over 15°. The color scheme in Figure 5c represents recrystallized grains (blue), recovered grains (yellow), and original grains (red). The proportion of LAGBs in Figure 5b is close to 90%, these being are primarily located around the boundaries of elongated grains. However, recrystallization is limited at this temperature with only a few scattered recrystallized grains observed (Figure 5c). The alloy is mainly in the recovery stage due to the insufficient recrystallization energy at this low temperature.

The IPF map of SRA-600 °C is depicted in Figure 5d. A significant amount of recrystallization occurs near the grain boundaries of deformed grains with very small recrystallized grains. In Figure 5e, there is a decreasing trend in the proportion of LAGBs to 60.8%. Almost no LAGBs were found inside the recrystallized grains, as the substructures within the large grains transformed into HAGBs during the annealing process. Compared to Figure 5c, the proportion of blue color in Figure 5f significantly increases with a recrystallization ratio of 54%. The microstructure of SRA-650 °C in Figure 5g is similar to that of SRA-600 °C, with slight differences in the distribution of GB maps and GOS maps. The alloy microstructure has 57.1% LAGBs and the recrystallization proportion is 59% under this condition. The higher annealing temperature provides more sufficient energy for recrystallization.

As the annealing temperature continues to rise, the proportion of fine equiaxed grains after recrystallization continues to increase. As shown in Figure 5j,m, fine recrystallized grains almost fill the entire field of view when the annealing temperature is raised to 700 °C and 750 °C. The proportion of LAGBs does not change significantly and is primarily distributed within a few larger grains. The recrystallization proportions are 76% and 70% in Figure 5l,o, respectively. The SRA-700 °C has undergone almost complete recrystallization and the recrystallized grains of SRA-750 °C have slightly grown in size. Therefore, the alloy experiences the most complete recrystallization after annealing at 700 °C.

**Figure 6:**
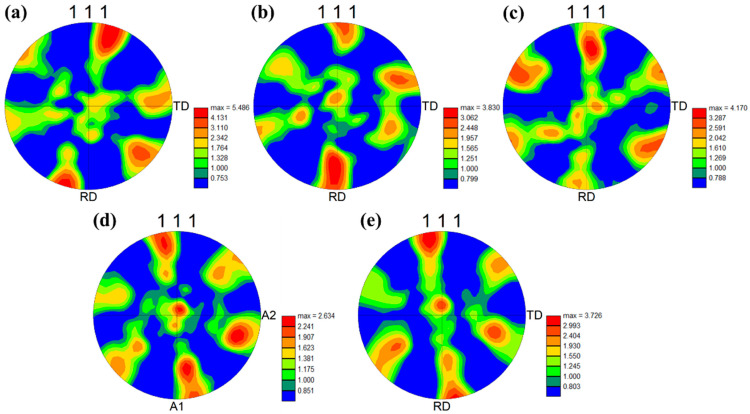
(111) Pole figures of the Au-15Ag-12Cu-6Ni alloy after annealing at different temperatures: (**a**) SAR-550 °C, (**b**) SAR-600 °C, (**c**) SAR-650 °C, (**d**) SAR-700 °C, (**e**) SAR-750 °C.

The textures of the cold-rolled alloy annealing at different temperatures were analyzed to study the influence of annealing temperature on the texture. The (111) pole figures of the alloy after annealing at different temperatures are presented in Figure 6. SAR-550 °C still exhibits a typical deformed structure, similar to the cold-rolled state, with no noticeable recrystallization. The texture characteristics remain almost identical to those of the cold-rolled state, primarily exhibiting a texture near the RD pole point. A deviation similar to that in Figure 3b has occurred, and the texture strength reaches a maximum of 5.486. Although partial recrystallization has occurred, the texture characteristics have remained almost unchanged in the 600 °C annealed samples. Notably, the deviated texture has returned to the RD pole point, possibly due to the appearance of recrystallized grains. The texture of SAR-650 °C is similar to that of SAR-600 °C, but the texture strength has increased. The SAR-700 °C undergoes complete recrystallization, resulting in a significant decrease in texture strength. Distinctively, the <111>//RD texture deviates in the opposite direction compared to Figure 3b. Furthermore, the second type of texture, located between the RD and TD pole points, gradually deviates towards the ND pole point. This evolution is attributed to the complete recrystallization of the alloy under this condition. As the temperature continues to rise into the grain growth stage, there is no change in the texture type, but the texture strength has increased.

**Figure 7:**
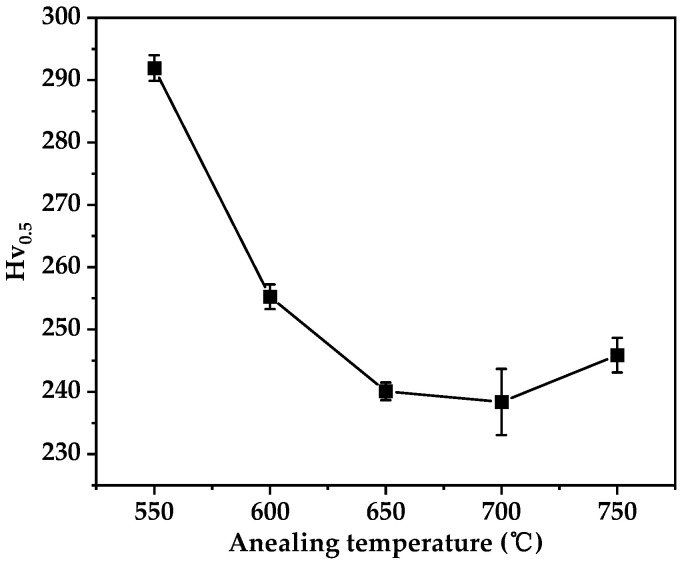
Hardness variation chart with annealing temperature for the Au-15Ag-12Cu-6Ni alloy.

The hardness variation of the alloy after annealing at different temperatures is depicted in Figure 7. It is evident that the alloy’s hardness gradually decreases with increasing annealing temperature, reaching its minimum value at 700 °C. At this temperature, stress relief annealing is most effective. Considering the trend of microstructural evolution after annealing, complete recrystallization occurs at an annealing temperature of 700 °C for 30 min. Therefore, annealing at 700 °C for 30 min is identified as the optimal stress-relief annealing condition.

### 3.3. Heat Treatment Process Optimization

#### 3.3.1. Annealing at Low Temperatures

In the Au-Ag-Cu-Ni alloy system, Au-Cu alloys can form ordered phases such as CuAu and Cu_3_Au, while Au-Ni alloys may undergo modulation decomposition. Both ordered phase transformations and modulation decomposition can significantly impact the alloy’s properties. The primary objective of this article is to enhance the alloy’s performance by controlling its microstructure through heat treatment. The focus is on optimizing the heat treatment regimen for the Au-15Ag-12Cu-6Ni alloy and investigating its effect on the alloy’s hardness. After stress relief annealing at 700 °C for 30 min, the sheet with a thickness of 3.4mm underwent cold rolling with a deformation of 66%. The direct heat treatment optimization for the as-processed sheet has been optimized. The heat treatment temperature ranges from 300 °C to 600 °C, with treatment times of 0.5~2 h.

**Figure 8:**
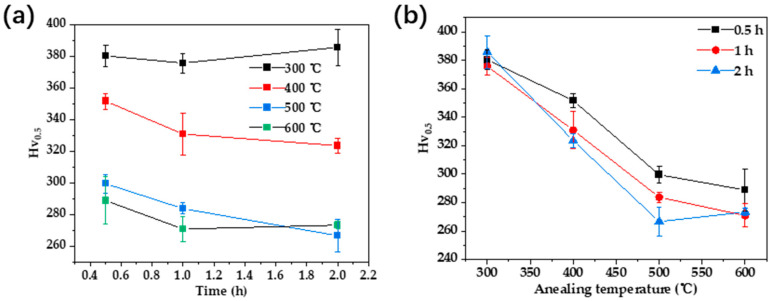
Alloy hardness variation chart with heat treatment regimen: (**a**) time; (**b**) temperature.

The samples subjected to different heat treatments were tested for hardness, and the results are presented in Figure 8. In Figure 8a, the heat treatment time exhibits varying trends in alloy hardness at different temperatures. At 300 °C, hardness initially decreases and then increases with prolonged heat treatment time. In the temperature range of 400 °C to 500 °C, there is a noticeable decrease in alloy hardness as the heat treatment time increases. At 600 °C, hardness initially decreases and then stabilizes with extended heat treatment time. Samples annealed at 400 °C exhibit significantly higher hardness than the hardness value after cold deformation, indicating that the Au-15Ag-12Cu-6Ni alloy still undergoes phase transformation. However, there are certain changes in the quantity or morphology of the phase transformation products, leading to a slight reduction in hardness compared to 300 °C.

When the heat treatment time is constant, the heat treatment temperature and the hardness of the alloy exhibit a similar trend. As the heat treatment temperature increases, the hardness of the alloy significantly increases, with a greater magnitude of change at low temperatures. The reduction in hardness change slows down as the temperature further increases. At a heat treatment temperature of 300 °C, the hardness reaches a maximum of 380 (Hv_0.5_), approximately 1.6 times that of the fully annealed state. The fact that the hardness of the alloy does not decrease but rather increases after heat treatment indicates a significant phase transformation at 300 °C. According to the previous literature review, the increase in hardness may be related to the ordered transformation in the Au-Cu alloy system [27,28].

#### 3.3.2. Double Heat Treatment

**Figure 9:**
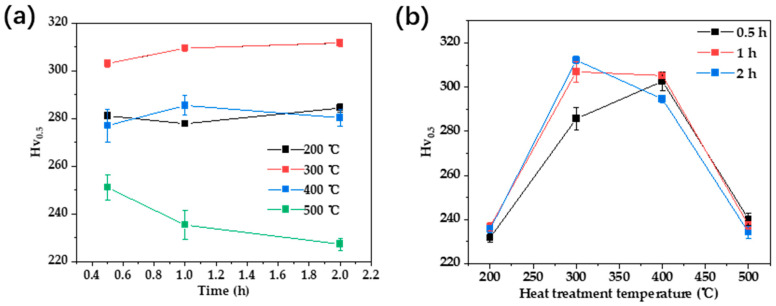
Alloy hardness variation chart with dual heat treatment regimen: (**a**) time, (**b**) temperature.

The alloy underwent annealing at 750 °C for 30 min followed by air cooling. Subsequently, a secondary heat treatment was conducted with temperatures ranging from 200 °C to 500 °C and treatment times of 0.5 h, 1 h, and 2 h, respectively. Hardness tests were conducted after the dual heat treatment, and the results are shown in Figure 9. Figure 9a shows that the hardness of the Au-15Ag-12Cu-6Ni alloy after double heat treatment changed relatively little with the treatment time. In Figure 9b, as the heat treatment temperature rose, the hardness of the alloy initially increased and then decreased with the highest hardness at 300 °C and 400 °C. At 300 °C to 400 °C, the highest hardness during heat treatment is related to the ordered transformation in the Au-Cu alloy system. During heat treatment below 400 °C, ordered transformation occurs, increasing the strength of the alloy. At temperatures above 400 °C, the strengthening phase dissolves back into the matrix, leading to a decrease in hardness. However, compared to the highest hardness of 380 (Hv_0.5_) obtained by low-temperature annealing, the strengthening effect of double annealing is slightly inferior. Therefore, the final heat treatment regimen is 300 °C/0.5 h.

### 3.4. Phase Analysis

**Figure 10:**
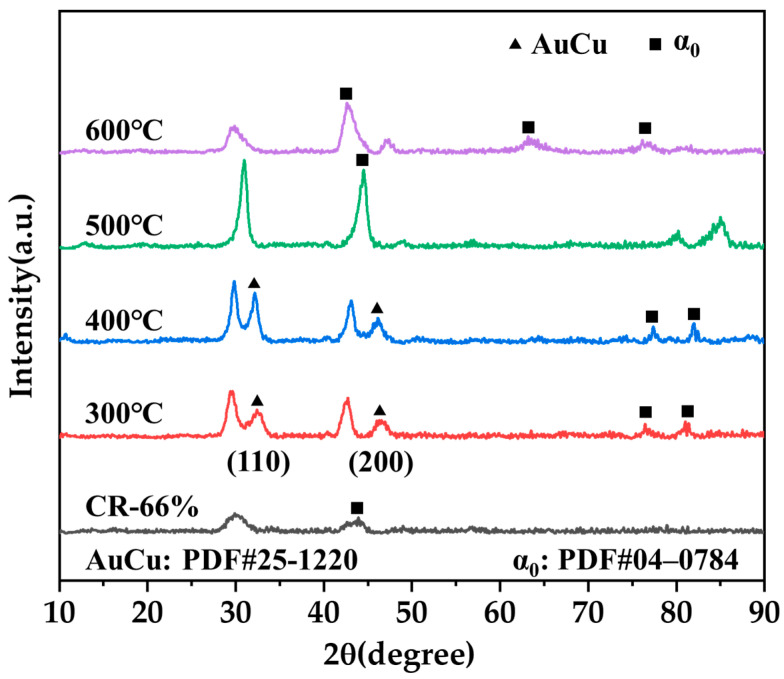
XRD spectra of the Au-15Ag-12Cu-6Ni alloy after heat treatment at different temperatures.

The optimal result of the heat treatment regimen is 300 °C/0.5 h. Additionally, HT-400 °C exhibits a high hardness, suggesting that the ordered phases enhance the alloy’s hardness. The XRD spectra of the Au-15Ag-12Cu-6Ni alloy after heat treatment at different temperatures (CR-66%, HT-300 °C/0.5 h, HT-400 °C/0.5 h, HT-500 °C/0.5 h, and HT-600 °C/0.5 h) are shown in Figure 10. It is evident that the alloy precipitated a substantial amount of ordered phases AuCu in HT-300 °C/0.5 h and HT-400 °C/0.5 h, explaining the significant increase in hardness at these temperatures.

**Figure 11:**
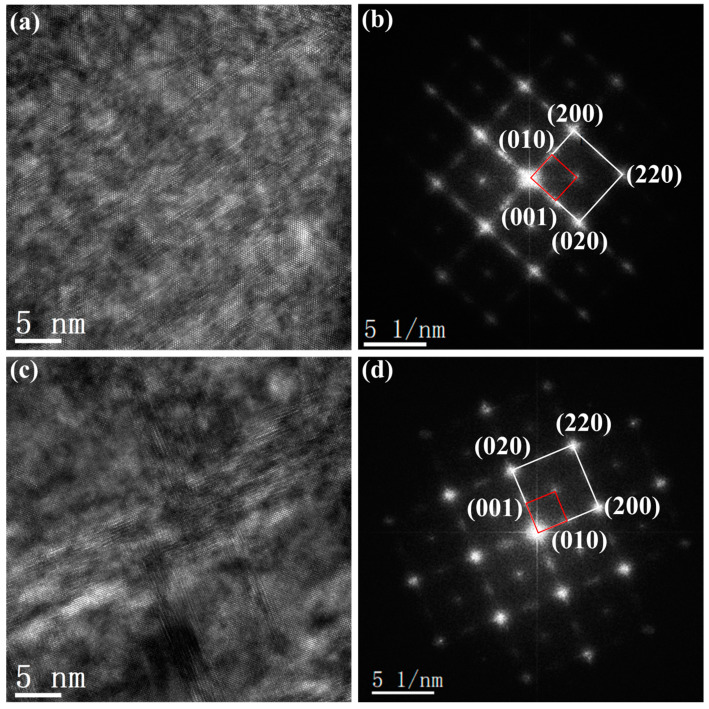
Bright filed images and the SAED pattern of (**a**,**b**) HT-300 °C/0.5 h and (**c**,**d**) HT-400 °C/0.5 h.

Figure 11 shows TEM images of HT-300 °C/0.5 h and HT-400 °C/0.5 h. The bright field images in Figure 11a,c correspond to HT-300 °C/0.5 h and HT-400 °C/0.5 h. Figure 11b,d were obtained from Figure 11a,c by fast Fourier transform (FFT) spectroscopy, respectively. Both selected area electron diffraction (SAED) patterns along the crystallographic band axis of [001]_FCC_ demonstrate the existence of gold-based solid phase and ordered phase AuCu. In Figure 11b,d, strong spots (white squares) represent the face-centered cubic structure (FCC) of the α_0_ matrix, while weak spots (red squares) indicate the ordered phase AuCu with a tetragonal structure. The SAED patterns effectively prove the existence of the ordered phase AuCu, the precipitation of the ordered phase is precisely what enhances the hardness of the alloy.

Through investigating the evolution of the microstructure and properties of the Au-15Ag-12Cu-6Ni alloy, the optional heat treatment regimen has been determined and the treated alloy exhibits a pleasing hardness of 380 (Hv_0.5_). The Au-15Ag-12Cu-6Ni alloy holds great potential to become the basis of a new generation of wear-resistant conductive rings. However, further research is needed to explore its electrical conductivity, corrosion resistance, and other comprehensive mechanical properties.

## 4. Conclusions

This article primarily focuses on the preparation of a new electrically conductive ring alloy, which consists of Au-15Ag-12Cu-6Ni. It conducts an in-depth analysis of the alloy’s microstructure and texture evolution during the cold rolling and annealing processes with hardness improvement as the main optimization objective for the cold rolling and annealing schedules of the alloy. The main conclusions are:

1. The maximum cold deformation limit of the alloy is 66% and the grains initially underwent deformation and were subsequently crushed into finer grains with the increase in cold rolling reduction. Significant work hardening occurred during the cold rolling process and the hardness reached its peak of 315 (Hv_0.5_) at a cold rolling reduction of 66%.
2. With the increase in annealing temperature the alloy’s recrystallization ratio gradually rose, reaching complete recrystallization at 700 °C. Correspondingly, the alloy exhibited the most pronounced softening effect with the lowest hardness at 700 °C.
3. The cold deformation texture of the alloy was mainly oriented along <111>//RD and has a deviation of 60° from the RD towards the TD. As the deformation increased, the texture strength gradually increased. After annealing, the alloy retained the texture type obtained during cold deformation, but there was a certain degree of deviation in the texture angles.
4. After a 300 °C heat treatment for half an hour, a substantial amount of ordered phases AuCu precipitated in the finished sheet, leading to significant strengthening of the alloy with a hardness of 380 (Hv_0.5_).

## Figures and Tables

**Table 1:** Chemical composition of different locations of the ingot (wt.%).

Position	Au	Ag	Ni	Cu
Upper section	65.56	15.81	6.36	12.27
Upper middle section	66.99	15.09	5.89	12.03
Lower middle section	66.36	15.79	5.8	12.05
Lower section	67.43	14.75	5.9	11.92

## Data Availability

Data are contained within the article.
